# Enhanced S-cone syndrome in a family: first long-term (12-year) follow-up and literature review

**DOI:** 10.1007/s00417-026-07300-6

**Published:** 2026-06-18

**Authors:** Silvia Pignatto, Asia Amelia Martin, Ana Ursula Gavric, Andrej Meglič, Enrico Redolfi De Zan, Paolo Lanzetta

**Affiliations:** 1 Department of Medicine - Ophthalmology, University of Udine, Piazzale Santa Maria della Misericordia, Udine, 33100 Italy; 2 Istituto Europeo di Microchirurgia Oculare (IEMO), Udine, Italy; 3 Eye Hospital, University Medical Centre Ljubljana, Ljubljana, Slovenia; 4 Faculty of Medicine, University of Ljubljana, Ljubljana, Slovenia

**Keywords:** Enhanced S-cone Syndrome, NR2E3, Inherited retinal dystrophy, Nyctalopia, Topical carbonic anhydrase inhibitors, Choroidal neovascularization

## Abstract

**Purpose:**

To describe the long-term clinical course of Enhanced S-cone Syndrome (ESCS) in three siblings carrying the same NR2E3 variant, and to report therapeutic outcomes of choroidal neovascularization (CNV) and cystic macular changes during a 12-year follow-up from early childhood.

**Methods:**

This is a retrospective observational family case with literature review. Three siblings were examined periodically over 12 years beginning in early childhood. Clinical evaluation included best corrected visual acuity (BCVA), cycloplegic refraction, multimodal imaging. CNV was treated with intravitreal ranibizumab, while cystic macular changes were managed with topical carbonic anhydrase inhibitors (CAIs).

**Results:**

The three siblings shared a homozygous splice site variant (c.119–2 A > C) in the NR2E3 gene and ERG showed ESCS pathognomonic features. In the eldest brother, acute onset esotropia secondary to visual impairment caused by CNV represented the first clinical manifestation. Following the onset of night blindness between ages 11 and 14, the older brother and sister developed macular cystic changes; however, topical CAIs were ineffective. Notably, they maintained stable visual acuity over a 12-year observation period.

**Conclusion:**

This family report underscores the clinical variability in ESCS and suggests that the visual acuity of young patients with ESCS remains stable, even in the presence of macular schisis. CNV was successfully treated with two intravitreal injections of ranibizumab, whilst topical CAIs proved ineffective for treating cystic patterns. Additional long-term studies are required to better understand the natural history of ESCS and improve therapeutic strategies.

## Key Messages

*****What is known***:**

- Enhanced S-cone Syndrome (ESCS) is a rare inherited retinal dystrophy caused by variants in the NR2E3 gene, characterized by variable clinical expression and early-onset night blindness.

*****What is new***:**

- This is the first long-term (12-year) family follow-up of three siblings carrying the same NR2E3 variant, documenting that visual acuity may remain stable from early childhood into young adulthood despite the development of nyctalopia and cystic macular changes.
- Choroidal neovascularization in ESCS can be successfully treated with intravitreal injections of ranibizumab, achieving long-term anatomical and functional stabilization, whereas macular cysts appear to be resistant to topical carbonic anhydrase inhibitor therapy.

## Introduction

ESCS is a rare, slowly progressive autosomal recessive inherited retinal degeneration caused by variants in NR2E3 gene and characterized by pathognomonic electrophysiological features [1]. NR2E3 gene encodes a ligand-dependent transcription factor which plays a key role for proper rod and cone photoreceptor differentiation and survival [2]. Loss of function variants cause the abnormal specialization of postmitotic photoreceptor precursor cells, misdirecting their cell-fate from rods to short-wavelength cones thus leading to an increase in the S-cone population [3, 4]. Postmortem histopathologic and immunocytochemical analysis of a 77-year-old donor eye with advanced ESCS showed a highly disorganized and degenerated retina. The sample was characterized by a lack of structurally recognizable rods and approximately twice the expected number of cones, with an abnormal ratio of S to L/M cones and twice the normal cone density in the central retina [3]. To date, at least 49 variants in the NR2E3 gene have been described resulting in ESCS phenotypes with variable expressivity, manifesting either in a homozygous or compound heterozygous state [1, 5].

Long-term follow-up on children and siblings diagnosed early with ESCS have not been extensively documented in the literature. This study presents a 12-year longitudinal follow-up of three siblings diagnosed with ESCS during early childhood.

## Clinical case

A., the older brother, came to our service in May 2011 at the age of five due to esotropia and visual loss in the right eye. His best corrected visual acuity (BCVA) was 20/80 in the right eye and 20/32 in the left eye. Cycloplegic refraction evidenced a hyperopic error in both eyes, while fundus examination identified a round-shaped pigmented fibrotic subretinal lesion along the superior arcade with serous retinal epithelium detachment and retinal exudates in his right eye. A similar fibrotic subretinal peripapillary lesion was found in his left eye. In association, pale white dots along the arcades were visible in both eyes. Optical coherence tomography (OCT) confirmed a hyperreflective lesion with subretinal fluid and intraretinal cystic changes in the right eye and a peripapillary hyperreflective lesion in the left eye without exudation. Fundus autofluorescence (FAF) highlighted multiple fine hyperautofluorescent foci along the arcades in both eyes. Fluorescein angiography revealed the presence of choroidal neovascularization in the right eye and retino-choroidal anastomosis in the left eye. Electroretinography showed normal rod-specific ERG and normal oscillatory potentials. Dark-adapted (DA) maximal response and light-adapted (LA) cone response were normal and had a similar waveform. The 30-Hz flicker was delayed with very low amplitude and full-field ERG demonstrated a significantly magnified S-cone signal.

In June 2011 the patient received in his right eye two intravitreal injections of ranibizumab over a 2-month period to prevent visual deterioration and to reduce the risk of deprivation amblyopia. The intravitreal anti-VEGF treatment led to an excellent outcome, resulting in resolution of subretinal fluid and improvement in BCVA to 20/32.

Contextually, we screened the patient’s siblings: his sister C. and brother G., who were four and two years old, respectively. At baseline, both patients were hyperopic and exhibited age-appropriate visual acuity. Fundus examination identified faint white dots along the arcades in both eyes and FAF imaging revealed multiple hyperautofluorescent spots along the arcades. The standard full-field ERGs (ffERG) of both siblings showed pathognomonic ESCS features: rod-specific response was undetectable; DA maximal response was reduced and had a similar waveform to the LA cone response; sensitivity to short wavelength light stimulus was increased. The 30-Hz flicker was severely reduced and delayed.

At the time of diagnosis, genetic testing of the three siblings was performed and a homozygous splice site variant c.119–2 A > C in the NR2E3 gene was identified, which both parents and the paternal grandmother carried heterozygously.

The patients were subsequently followed over a 12-year period and ERGs were repeated in accordance with the standards and protocols of the International Society for Clinical Electrophysiology of Vision (ISCEV) [6, 7]. An HK loop electrode was placed in the lower eyelid fornix, while a silver chloride reference electrode was positioned on the ipsilateral temple and the ground electrode on the forehead. Pupils were dilated using 1% tropicamide (Mydriacyl, Alcon). Full-field ERGs were recorded using a Ganzfeld ColorDome flash stimulator (Diagnosys LLC). Dark-adapted ERGs were obtained after 20 min of dark adaptation, and light-adapted ERGs after 10 min of light adaptation at a background luminance of 30 cd/m². The S-cone ERG was elicited using 0.01 cd·s/m² blue (449 nm) stimuli on a 140 cd/m² amber (594 nm) background.

Electrodiagnostic testing revealed pathognomonic ERG features consistent with enhanced S-cone syndrome in all three subjects [1, 8–11]. The DA 0.01 ERG was undetectable and the oscillatory potentials were poorly defined and barely above the noise level. The DA 3 and DA 10 ERGs were reduced in amplitude and delayed. The 30 Hz flicker ERG was delayed and markedly reduced, with an amplitude lower than the a-wave of the LA 3 ERG. The LA 3 ERG was also delayed with a reduced b-wave amplitude. Both DA 3 and LA 3 ERGs showed simplified waveforms of comparable shape. The S-cone ERG demonstrated increased amplitude with delayed latency.

**Fig. 1:**
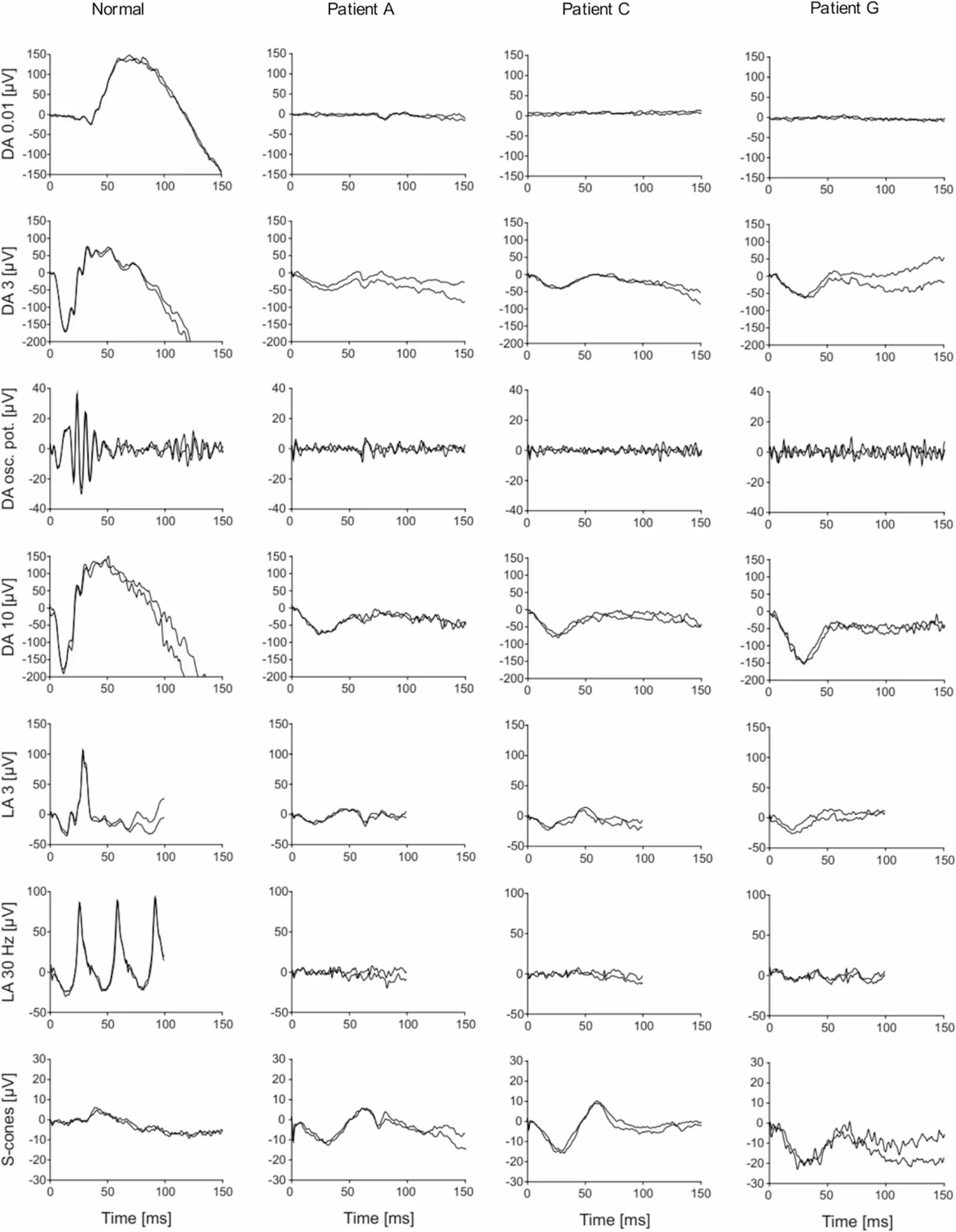
Full-field ERG responses recorded according to ISCEV standards in a healthy control and for the three patients

Full-field ERG values and responses recorded according to ISCEV standards for the three patients were reported in Table 1e Fig. 1.

**Table 1:** Full-field ERG values for the three patients. Values presented in regular font are within the laboratory reference range, underlined values indicate slight deviations, while underlined and bold values indicate significant deviations, - denotes absent wave

			Normal values (range)	Patient A		Patient C		Patient G	
				Re	Le	Re	Le	Re	Le
**ffERG amplitude**	DA 0.01 ERG		99.0–201.8	**0**	**0**	**0**	**0**	**0**	**0**
	DA 3 ERG	a	89.5–160.4	**51.7**	**47.8**	**34.6**	**37.2**	**52.0**	**62.7**
		b	137.5–260.7	**45.7**	**38.1**	**29.7**	**37.7**	**44.0**	**68.0**
	DA 10 ERG	a	106.0–192.0	**88.7**	**83.3**	**66.0**	**72.2**	**93.0**	**147.8**
		b	150.8–267.3	**68.0**	**64.0**	**61.9**	**63.2**	**61.6**	**114.8**
	DA osc. pot.		18.8–41.0	**3.8**	**3.2**	**2.0**	**1.5**	**8.0**	**4.9**
	LA 3 ERG	a	14.0–32.2	16.3	15.8	17.9	19.0	17.0	24.7
		b	65.0–149.9	**21.6**	**23.8**	**30.2**	**32.6**	**20.3**	**29.0**
	LA 30 Hz flicker		53.9–116.5	**16.7**	**7.6**	**6.3**	**9.5**	**11.2**	**12.8**
	S-cones		1.9–8.5	**15.5**	**17.1**	**18.9**	**24.6**	**13.8**	**17.0**
**ffERG peak time**	DA 0.01 ERG		80.0–110.4	**-**	**-**	**-**	**-**	**-**	**-**
	DA 3 ERG	a	14.0–21.1	**32.0**	**31.0**	**26.5**	**26.0**	**30.5**	**31.0**
		b	32.9–45.9	**56.5**	**56.5**	**72.5**	**58.5**	**54.0**	**57.0**
	DA 10 ERG	a	11.0–14.0	**27.5**	**23.0**	**26.5**	**26.0**	**30.0**	**29.5**
		b	30.0–53.3	**71.5**	**76.0**	**72.5**	**67.0**	**63.0**	**58.5**
	DA osc. pot.		22.0–24.0	*21.0*	*20.5*	**27.0**	**27.0**	*21.0*	*21.0*
	LA 3 ERG	a	13.0–15.0	**19.5**	**19.5**	**17.0**	**18.5**	**16.0**	**19.5**
		b	27.9–31.0	**49.0**	**46.0**	**48.5**	**49.0**	**46.0**	**47.0**
	LA 30 Hz flicker		25.0–28.0	**38.5**	**41.0**	**42.5**	**43.0**	**37.5**	**40.0**
	S-cones		38.0–51.0	**61.0**	**60.5**	**61.5**	**60.5**	**65.0**	**63.0**

**Fig. 2:**
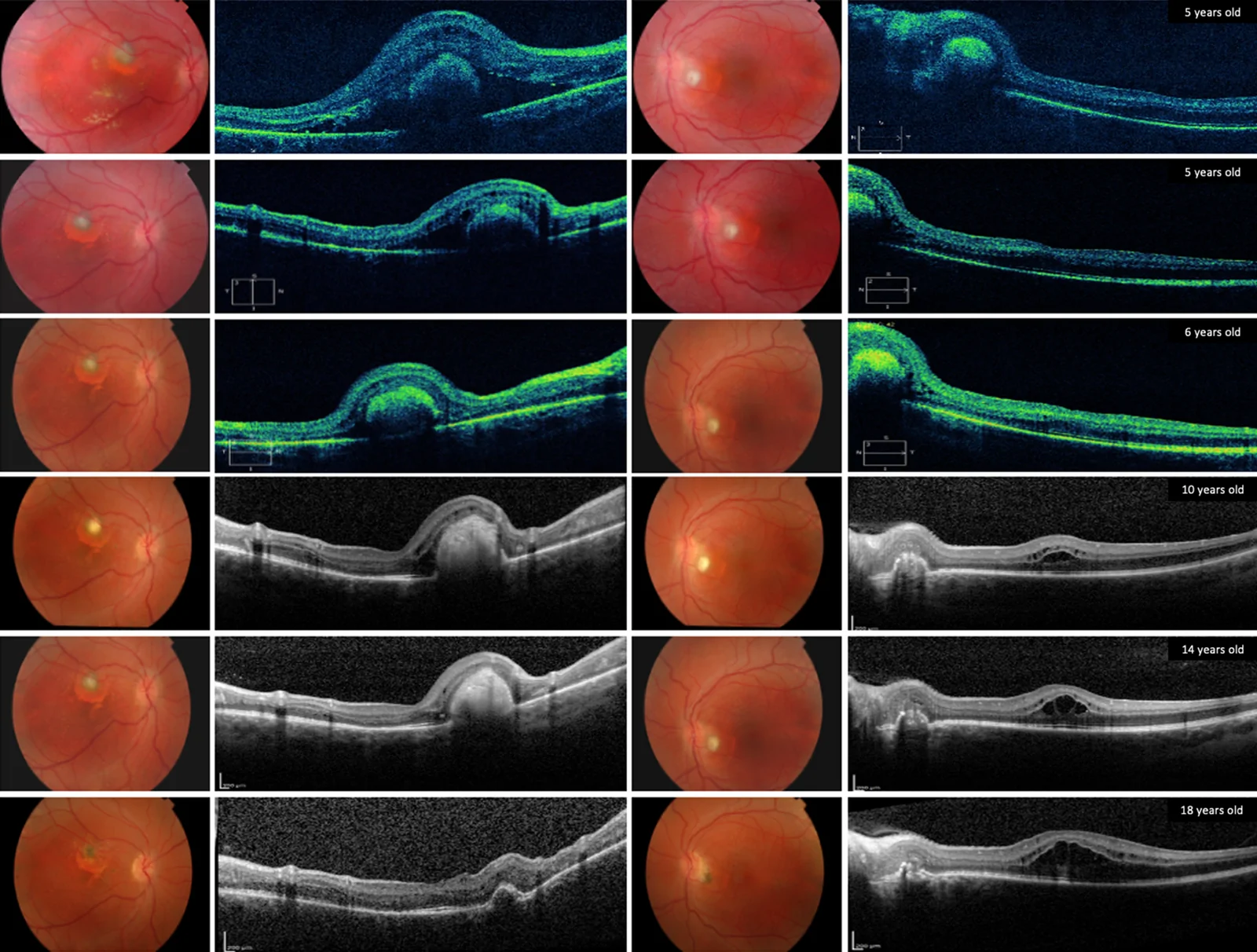
Multimodal imaging of patient A. Color fundus photography and OCT illustrate the progression of choroidal neovascularization in both eyes, with the lesions evolving into fibrotic subretinal lesions. In the left eye, intraretinal cystic changes developed progressively over the follow-up period

Patient A showed progressive involution of the bilateral subretinal fibrotic lesions, with no evidence of recurrent exudation over the follow-up. At the age of 14, he reported the onset of night blindness and clinical examination revealed schisis-like maculopathy characterized by intraretinal cystic changes. Therefore, treatment was started with topical CAI in the left eye, with no significant response. At the age of 18, BCVA was 20/22 in the right eye and 20/32 in the left eye. Figure 2; Table 2 summarized clinical history and multimodal imaging of patient A.

**Table 2:** Clinical history and longitudinal follow-up of patient A. Summary of BCVA, refractive status, and key clinical features recorded throughout the follow-up period

Age	Follow up	Best Corrected Visual Acuity (BCVA)	Refraction	Features/History
5	Baseline	RE: 20/80 LE: 20/32	+ 6.50 sf both eyes	Hyperautofluorescent spots; CNV
5	6 months	RE: 20/32 LE: 20/25	+ 6.50 sf both eyes	A reduction in subretinal fluid was observed two months after the second injections of ranibizumab
6	1 year	RE: 20/32 LE: 20/25	+ 6.50 sf both eyes	
10	5 years	RE: 20/25 LE: 20/22	+ 6.50 sf both eyes	Macular schisis in the LE
14	9 years	RE: 20/22 LE: 20/22	+ 6.50 sf both eyes	Night blindness; Macular schisis in the LE
18	13 years	RE: 20/22 LE: 20/32	+ 6.50 sf both eyes	Atrofic scar in the RE; Macular schisis in the LE

**Fig. 3:**
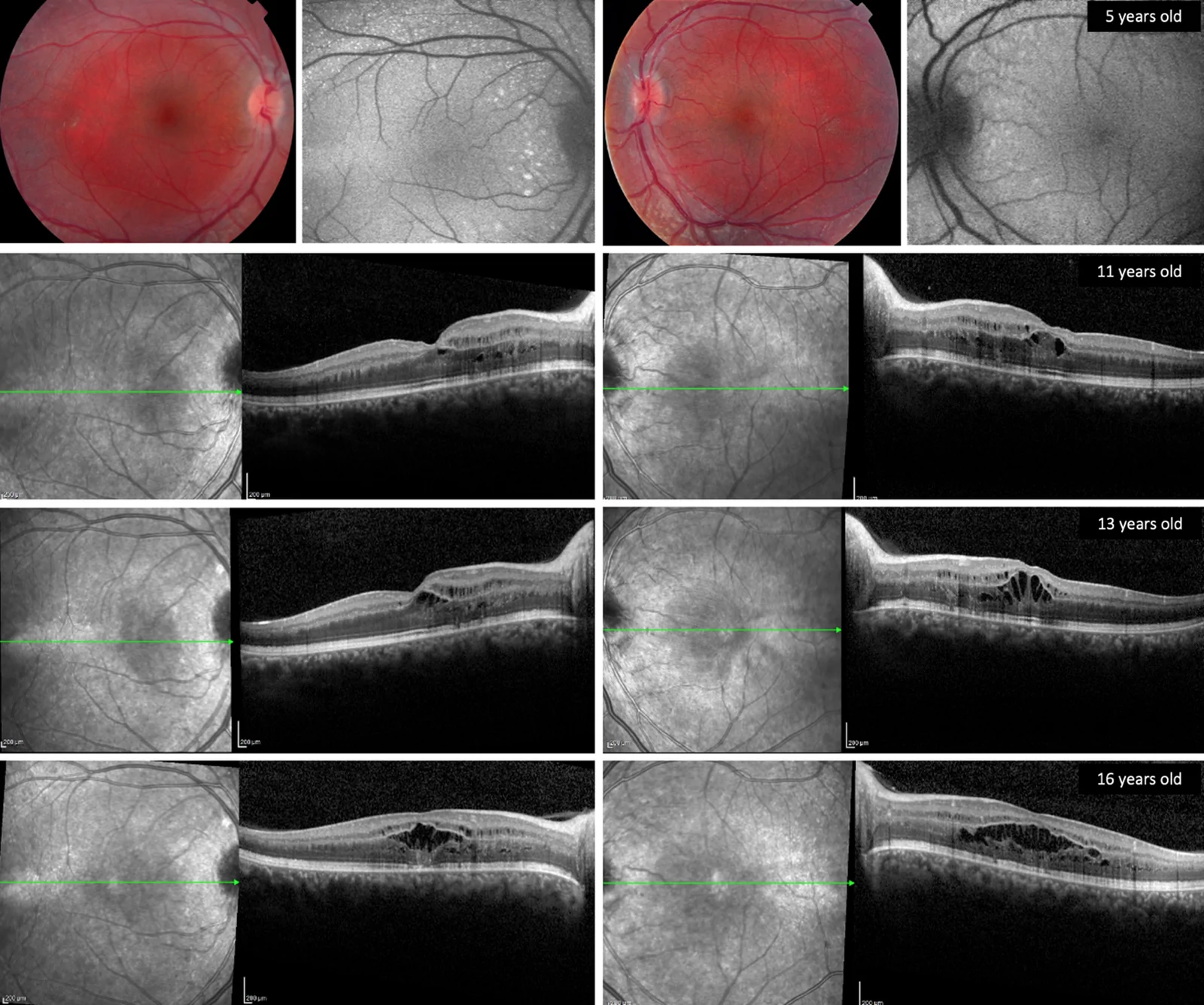
Multimodal imaging of patient C. Baseline color fundus photography of both eyes shows grayish appearance along the arcades, and FAF show hyperautofluorescent spots located peripheral, inside the vascular arcades. Serial OCT scans of both eyes demonstrate a progressive increase in intraretinal cystic changes over the follow-up period

Patient C showed no significant changes until the age of 11, when she reported the onset of night blindness. At that time, bilateral cystic macular changes were noted for the first time. Consistent with the findings in her brother, treatment with topical CAI was initiated in both eyes but proved ineffective. Over the subsequent years, a progressive worsening of the foveoschisis was observed. By the age of 16, her BCVA had slightly decreased to 20/25 bilaterally. Figure 3; Table 3 summarized clinical history and multimodal imaging of patient C.

**Table 3:** Clinical history and longitudinal follow-up of patient C. Summary of BCVA, refractive status, and key clinical features recorded throughout the follow-up period

Age	Follow up	Best Corrected Visual Acuity (BCVA)	Refraction	Features
5	Baseline	RE: 20/20 LE: 20/20	+ 4.50 sf both eyes	FAF: Hyperautofluorescent spots
11	6 years	RE: 20/20 LE: 20/20	+ 3.75 sf both eyes	Night blindness; Macular schisis in both eyes
13	8 years	RE: 20/20 LE: 20/20	+ 3.75 sf both eyes	Macular schisis in both eyes
16	12 years	RE: 20/25 LE: 20/25	+ 3.50 sf both eyes	Macular schisis in both eyes

**Fig. 4:**
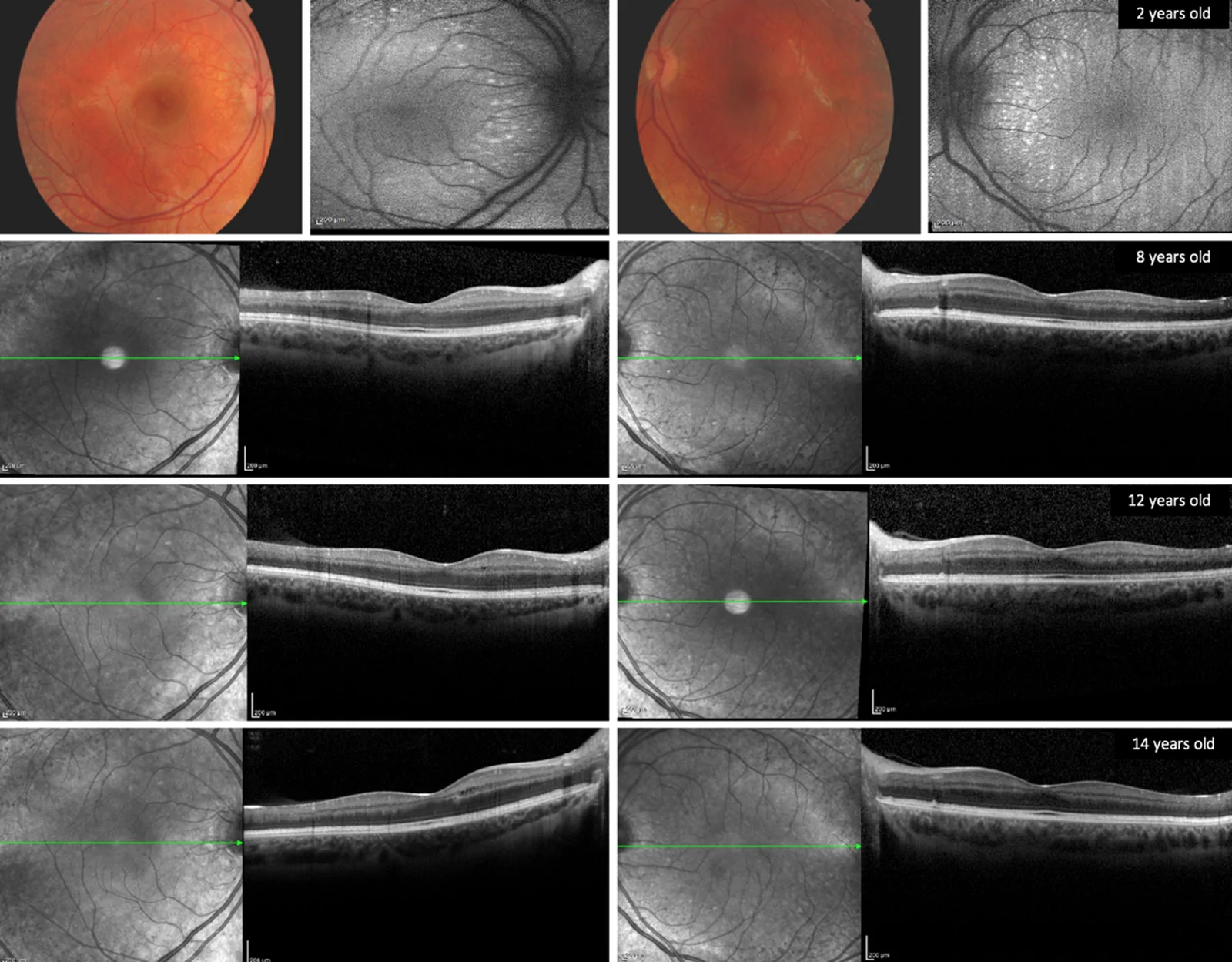
Multimodal imaging of patient G. Baseline color fundus photography of both eyes shows grayish appearance along the arcades, and FAF show hyperautofluorescent spots located peripheral, inside the vascular arcades. Serial OCT scans demonstrate stable macular architecture in both eyes over the follow-up period. A small intraretinal cyst was first observed in the right eye at 12 years of age

Patient G, the youngest brother, had a BCVA of 20/20 in both eyes. He reported the onset of night blindness at the age of 12, when a small cyst was found in the right eye. Despite this finding, his BCVA remained stable. Figure 4; Table 4 summarized clinical history and multimodal imaging of patient G.

**Table 4:** Clinical history and longitudinal follow-up of patient G. Summary of BCVA, refractive status, and key clinical features recorded throughout the follow-up period

Age	Follow up	Best Corrected Visual Acuity (BCVA)	Refraction	Features/History
2	Baseline	RE: 20/20 LE: 20/20	+ 3.50 sf both eyes	FAF: Hyperautofluorescent spots
8	6 years	RE: 20/20 LE: 20/20	+ 1.00 sf both eyes	
12	10 years	RE: 20/20 LE: 20/20	+ 1.00 sf both eyes	Night blindness; cystic changes in the RE
14	12 years	RE: 20/20 LE: 20/20	+ 1.00 sf both eyes	Cystic changes in the RE

## Discussion

To our knowledge, this is the only report describing the clinical course of ESCS in three siblings from early childhood through 12 years of follow-up, thereby providing insight into the natural history of this rare condition.

ESCS typically presents with nyctalopia in the first decade of life, with or without reduced visual acuity and visual field abnormalities. However, in young children, the disease may manifest with indirect signs, such as hyperopia and accommodative esotropia. Bilateral involvement is consistently observed, and color vision may be preserved in the early stages. In our cohort, the eldest sibling initially presented with acute onset esotropia, whereas nyctalopia developed between 11 and 14 years of age, with no impairment of color vision.

Despite sharing the same NR2E3 variant and distinct phenotypic presentations, they maintained a stable visual acuity over more than a decade. This is consistent with the well-documented intra- and interfamilial variability reported in the literature [1]. Retinal dysfunction pattern is a common finding among ESCS patients, but the amount of retinal degeneration is widely variable. Consequently, the same genotype can exhibit heterogeneous clinical manifestations in different patients, varying from slight retinal changes in the absence of any visual symptoms to high hyperopia associated with sub-retinal fibrosis [12]. It has been hypothesized that the wide range of clinical phenotypes observed in patients affected by ESCS may be the consequence of complex interactions involving transcriptional coregulators and genetic modifiers [3].

Reports describing ESCS presentation in children are limited. Few studies describing the pediatric phenotype in ESCS reported that most children initially lack the typical deep nummular pigmentation and that fundus examination findings may be normal or showing subtle RPE changes along the arcades [13, 14]. In adults, fundus examination typically reveals pigmentary changes of the retinal pigment epithelium (RPE), primarily along the vascular arcades or in the mid-peripheral area that may be associated, in more advanced cases, with rounded pigment accumulations. More specifically, macular involvement may include cystic changes and schisis. Additional features such as vitreous degeneration, chorioretinal atrophy, peripheral retinoschisis and posterior subcapsular cataract have also been reported [8]. Interestingly, in early childhood we observed subretinal white dots along the arcades which are usually described as an ESCS feature potentially indicative of an early disease stage [13]. Later, all siblings developed intraretinal macular cysts, consistent with findings in adult ESCS [8]. The youngest patient presented at 5 years old with choroidal neovascularization (CNV) and subretinal fibrosis, complications that have been previously reported in ESCS [15–17].

ERG findings are pathognomonic and allow diagnosis. Typical ERG features include undetectable rod-specific response; relatively delayed responses of similar waveforms to the same intensity flash under both scotopic and photopic conditions; a-wave amplitude of the transient photopic cone ERG larger than the amplitude of the photopic 30-Hz flicker cone ERG; b-wave in the transient photopic cone ERG large and prolonged in contrast to the peaking b-wave in healthy subjects; remarkable increase in S-cone-specific ERG testing and significantly reduced L- and M-cone function [8–11].

FAF and OCT have been a useful supplement in the diagnostic process and in monitoring the disease progression, as previously described [18].

Several treatments have been used to manage associated complications, and few studies have suggested the efficacy of topical CAIs and oral acetazolamide in the treatment of cystic macular lesions, leading to a decrease in central macular thickness, an increase in retinal sensitivity, and improvement in visual acuity [19–21]. Hajali and Fishman reported the efficacy of topical dorzolamide for the management of macular cysts in a 12-year-old patient [22]. The presumed mechanism of action of CAIs is their ability to enhance RPE pumping function by inducing an acidification of the subretinal space, a decrease of the standing potential as well as an increase in retinal adhesiveness. Immunocytochemical analysis performed on human retina demonstrated a particular enrichment of carbonic anhydrase XIV on Muller cells, astrocytes and RPE membranes, that is thought to be responsible for pH and volume buffering in retinal neurons and their surrounding extracellular matrix. These findings suggested that CA XIV might be the target of CAIs resulting in subretinal fluid absorption enhancement [23].

No therapeutic effect was observed with topical dorzolamide in the two patients treated in our report. In accordance with cases of CAIs non-responder patients previously reported by Bušić et al., these findings suggest that a deficiency in cell-to-cell adhesion could drive the pathogenetic process rather than retinal barrier breakdown itself [24]. Bušić et al. also described a marked difference in treatment response to CAIs between adults and children and suggested that the breakdown of the blood-retinal barrier may play a more important role later in life [24]. Some authors have also hypothesized that the effectiveness of CAIs may also depend on the residual function of the RPE cells in individual patients [25, 26].

The association between CNV and ESCS is already extensively reported [17, 27, 28], however the pathological pathways have not been thoroughly explored. In retinal dystrophies photoreceptor dysfunction, RPE changes and choriocapillaris damage may produce weakness zones through which choroidal vessels can grow. Since cones are known to have a higher oxygen demand than rods, it has also been described that in ESCS patients the increased number of cones might induce an ischemic condition and the consequent upregulation of VEGF production, promoting neovascularization [29, 30]. CNV in ESCS patients can occur early in childhood and seems to have a high possibility of spontaneous regression, leaving a circumscribed subretinal fibrosis [15, 17].

The most common presentation of CNV is acute central vision loss, while in pediatric population indirect signs may represent the initial manifestations [28, 31]. Accordingly our patient suffered from acute onset esotropia as a consequence of vision impairment. Untreated CNV may lead to persistent visual loss due to the development of fibrotic subretinal scarring and/or deprivation amblyopia. To prevent visual deterioration and reduce the risk of amblyopia, patient A was treated with two intravitreal injections of ranibizumab. The efficacy of both bevacizumab and ranibizumab in treating pediatric CNVs has been reported in the last decades, leading to favorable outcomes in clinical practice [32]. These findings suggest that the use of intravitreal anti-VEGF drugs could prevent permanent visual loss secondary to CNV in ESCS.

Further investigation into ESCS etiopathogenesis is needed to fully understand the underlying molecular pathways, signaling networks and to identify the role of modifier genes for the development of effective target therapies. Possible strategies might include gene therapy by viral NR2E3 gene transfer, modulation of NR2E3 mutant protein activity by pharmacological agents, and differential activation of potential modifier genes [33].

## Conclusion

To our knowledge, this is the only family case report with a long observational period, offering a perspective on the natural progression from childhood into early adulthood. This family report highlights the phenotypic variability that characterizes ESCS, even when caused by the same variant and within the same family. Young patients may maintain stable visual acuity despite heterogeneous clinical presentations and complications. Intravitreal anti-VEGF injections are effective in the treatment of CNV, whereas topical carbonic anhydrase inhibitors did not show therapeutic benefit.

## Data Availability

No datasets were generated or analysed during the current study.
